# *Clostridium difficile* Infection and Proton Pump Inhibitor Use in Hospitalized Pediatric Cystic Fibrosis Patients

**DOI:** 10.1155/2011/345012

**Published:** 2011-11-15

**Authors:** John F. Pohl, Raza Patel, Jeffery T. Zobell, Ellen Lin, E. Kent Korgenski, Kody Crowell, Mark W. MacKay, Aleesha Richman, Christian Larsen, Barbara A. Chatfield

**Affiliations:** ^1^Department of Pediatric Gastroenterology, Hepatology, and Nutrition, Primary Children's Medical Center, University of Utah School of Medicine, Salt Lake City, UT 84113-1103, USA; ^2^Pharmacy Department, Primary Children's Medical Center, Salt Lake City, UT 84113-1100, USA; ^3^Department of Pediatrics, Primary Children's Medical Center, University of Utah School of Medicine, Salt Lake City, UT 84113-1100, USA; ^4^Pharmacy Department, MaKay-Dee Hospital Center, Intermountain Healthcare, Ogden, UT 84403, USA; ^5^Department of Pediatric Pulmonology, Primary Children's Medical Center, University of Utah School of Medicine, Salt Lake City, UT 84158, USA

## Abstract

Children with cystic fibrosis (CF) often take proton pump inhibitors (PPIs), which helps improve efficacy of fat absorption with pancreatic enzyme replacement therapy. However, PPI use is known to be associated with *Clostridium difficile*-(*C. diff*-) associated diarrhea (CDAD). We retrospectively evaluated the incidence of *C. diff* infection from all pediatric hospital admissions over a 5-year period at a single tertiary children's hospital. We found significantly more *C. diff*-positive stool tests in hospitalized patients with CF compared to patients with no diagnosis of CF. However, use of a PPI was not associated with an increased risk of CDAD in hospitalized CF patients. In summary, *C. diff* infection is more common in hospitalized pediatric CF patients although PPI use may not be a risk factor for CDAD development in this patient population.

## 1. Introduction

Cystic fibrosis (CF) is a hereditary multiorgan disease that affects approximately 30,000 people in the United States [1]. Mutation in the *cystic fibrosis transmembrane regulator (CFTR)* gene results in impaired ion transport within epithelial cells and leads to mucosal obstruction of exocrine glands, such as in the pancreas [1]. Subsequent hyperviscosity of pancreatic enzyme secretions obstructs the pancreatic ducts and lead to pancreatic fibrosis with poor fat and protein absorption. As a result, CF patients receive pancreatic enzyme replacement therapy (PERT) to aid in fat absorption. Proton pump inhibitors (PPIs) can improve fat absorption and decrease symptoms of steatorrhea in CF patients by inhibiting breakdown of PERT by gastric acid [2, 3]. Therefore, PPI usage is widely used in the CF population [2–5]. 

Chronic antibiotic use, prolonged hospitalization, and acid-suppressive agents (such as PPIs) are risk factors for developing infection with *Clostridium difficile* (*C*. *diff*) [6, 7]. *C*. *diff* can manifest from asymptomatic colonization of the gastrointestinal tract to life-threatening conditions such as pseudomembranous colitis and toxic megacolon [6]. Gastric acidity protects the host against ingested pathogens, and this protection is impaired when acid production is suppressed [7]. Several studies have demonstrated that acid suppression therapy with PPIs may increase the risk of *C. diff*-associated diarrhea (CDAD) [8–12]. We retrospectively evaluated the prevalence of *C. diff* infection in the pediatric CF population at a single pediatric tertiary medical center and tried to determine if use of PPI increased the risk of infection in this patient population.

## 2. Methods

A retrospective chart review occurred over a 5-year period (January 1, 2005, through December 31, 2010) to determine how many tests had been performed for *C. diff* during all inpatient admissions. This data was obtained from the Intermountain Electronic Data Warehouse, Intermountain Primary Children's Medical Center electronic medical record and microbiology lab data. Testing for *C. diff* occurred by toxin ELISA assay or by toxin DNA analysis (Illumigene, Meridian Bioscience, Inc., Cincinnati). Those patients with CF who had a positive *C. diff* stool test during a hospital admission were further analyzed using the Cystic Fibrosis Foundation Patient Registry and the electronic medical record to determine if they had been on a PPI during this admission. Chi-square testing was used to compare prevalence of *C. diff* in hospitalized patients with and without CF, and relative risk analysis was used to determine the risk of *C. diff* in CF patients receiving a PPI [13]. The project was approved by the University of Utah Investigational Review Board.

## 3. Results

During the 5-year study period, 8543 tests for *C. diff* were performed on hospitalized patients (8302 non-CF controls and 241 CF cases). The prevalence of *C. diff* in controls versus CF patients was statistically significant (*P* < 0.05) with the period prevalence of *C. diff* infection being 20% (*n* = 1659) and 35.7% (*n* = 86), respectively. We were only able to determine the total number of *C. diff* tests that occurred, and testing may have occurred more than once in some patients (Table 1).

Next, we evaluated all CF patients who were hospitalized during this time period using the Cystic Fibrosis Foundation Patient Registry to determine which patients were on a PPI. We found 215 total patients which were different than the total number of patients found using the electronic medical record (241 patients). Of these 215 patients, 140 were on no PPI and 57 of these patients (40.7%) had CDAD. There were 75 patients who were on a PPI, and 30 patients (40%) had CDAD. A relative risk analysis was not significant suggesting that the risk of developing CDAD in CF patients was equal regardless of PPI exposure (Table 2).

## 4. Discussion

This retrospective study evaluated both the prevalence of *C. diff* infection in CF pediatric patients during inpatient admission compared to patients without CF and the risk of CDAD in CF patients receiving a PPI. We found that significantly more patients had positive *C. diff* testing during hospital admission with CF compared to non-CF patients. Unlike CF pediatric patients, the typical hospitalized patients with CDAD in the United States hospitals are elderly, female, and have associated sepsis [14]. Antibiotic use is also associated with CDAD in hospitalized patients [15].

It is known that the asymptomatic carriage rate of *C. diff* in CF can be as high as 50%, and associated factors leading to CDAD in hospitalized CF patients potentially could include risk factors such as recurrent hospitalization, prolonged intravenous antibiotic use, and lack of colonization with *Lactobacillus* species and other bacteria that may have inhibitory effects on the growth of *C. diff *[16–18]. Other diseases associated with *C. diff*, such as Crohn's disease, occur more frequently in CF patients [19]. Additionally, CF genotypes, such as N1303K, are associated with more severe clinical presentations of CDAD [20]. 

PPI use has been associated with an increased risk of CDAD, especially in the elderly [21]. The effect is presumable due to gastric acid inhibition by PPIs, and the risk of infection is increased with escalating PPI dosing [22]. Interestingly, we saw no increased incidence of CDAD in CF pediatric patients, regardless of PPI usage status. The cause of this finding is unknown. A prior study looking at the risk of developing CDAD in a community setting did not find PPI usage associated with an increased risk of CDAD; however, other proposed risk factors for disease transmission in this population included person-to-person transmission and remote health care exposures [23]. Bacterial overgrowth is not always associated with intestinal inflammation in some CF patients, suggesting that there may be other microbiome, genetic, or inflammatory pathways that may explain our finding [24]. It is the clinical practice at our institution to place all CF patients on Lactobacillus GG at time of admission and throughout the intravenous antibiotic course. This treatment is not typically continued upon completion of an antibiotic treatment unless the patient has been diagnosed with CDAD during hospitalization. However, it is not known if such therapy is effective in preventing CDAD in this patient population [25]. 

There are weaknesses in this study. This study is retrospective and consists of data from a single tertiary medical center. In our review of the electronic medical record of all pediatric inpatient admissions, we could not determine if testing for *C. diff* occurred because of CDAD, although asymptomatic hospitalized patients presumably would not be tested for this pathogen. However, we were able to determine CDAD in hospitalized CF patients using the Cystic Fibrosis Foundation Patient Registry. Finally, we did not evaluate for other potential causes that would explain the lack of difference in CDAD regardless of PPI use in the CF population, although we did evaluate a large number of pediatric CF inpatient admissions over a 5-year period of time.

## 5. Conclusion

We found significantly more *C. diff* infections in hospitalized pediatric patients with CF compared to patients hospitalized for other medical reasons. PPI use by CF patients appears not to be associated with an increased risk of developing CDAD. We encourage judicious use of antibiotics as well as simple techniques in preventing disease transmission, such as good handwashing to prevent CDAD in CF pediatric patients. We recommend a prospective, multicenter study to corroborate these findings and to determine what specific risk factors in the CF pediatric population predispose to *C. diff* infection such as CF genotype, length of PPI usage, patient age, and presence of other comorbidities such as inflammatory bowel disease.

## Figures and Tables

**Table 1:** Positive *C. diff* tests for inpatient pediatric patients.

Total tests	8543
All tests in patients with no CF	8302
Positive tests in patients with no CF	1659 (20%)*
All tests in CF patients	241
Positive tests in patients with CF	86 (35.7%)*

*P < 0.05.

**Table 2:** CDAD in CF patients with/without PPI use.

Total CF patients admitted to hospital	215
CF patients on PPI and CDAD	30 (40%)*
CF patients on PPI and no CDAD	45
CF patients on no PPI and CDAD	57 (40.7%)*
CF patient on no PPI and no CDAD	83

*Relative risk 0.98 (95% confidence interval 0.698 to 1.382), *P* = 0.91.
